# Stress granules, RNA-binding proteins and polyglutamine diseases: too much aggregation?

**DOI:** 10.1038/s41419-021-03873-8

**Published:** 2021-06-08

**Authors:** Adriana Marcelo, Rebekah Koppenol, Luís Pereira de Almeida, Carlos A. Matos, Clévio Nóbrega

**Affiliations:** 1 Algarve Biomedical Center Research Institute (ABC-RI), Faro, Portugal; 2 PhD Program in Biomedial Sciences, Faculty of Medicine and Biomedical Sciences, University of Algarve, Faro, Portugal; 3 Centre for Biomedical Research (CBMR), Universidade do Algarve, Faro, Portugal; 4 Center for Neuroscience and Cell Biology (CNC), University of Coimbra, Coimbra, Portugal; 5 Faculty of Medicine and Biomedical Sciences, University of Algarve, Faro, Portugal; 6 Faculty of Pharmacy, University of Coimbra, Coimbra, Portugal; 7 Champalimaud Research Program, Champalimaud Center for the Unknown, Lisbon, Portugal

**Keywords:** Neuroscience, Neurological disorders

## Abstract

Stress granules (SGs) are membraneless cell compartments formed in response to different stress stimuli, wherein translation factors, mRNAs, RNA-binding proteins (RBPs) and other proteins coalesce together. SGs assembly is crucial for cell survival, since SGs are implicated in the regulation of translation, mRNA storage and stabilization and cell signalling, during stress. One defining feature of SGs is their dynamism, as they are quickly assembled upon stress and then rapidly dispersed after the stress source is no longer present. Recently, SGs dynamics, their components and their functions have begun to be studied in the context of human diseases. Interestingly, the regulated protein self-assembly that mediates SG formation contrasts with the pathological protein aggregation that is a feature of several neurodegenerative diseases. In particular, aberrant protein coalescence is a key feature of polyglutamine (PolyQ) diseases, a group of nine disorders that are caused by an abnormal expansion of PolyQ tract-bearing proteins, which increases the propensity of those proteins to aggregate. Available data concerning the abnormal properties of the mutant PolyQ disease-causing proteins and their involvement in stress response dysregulation strongly suggests an important role for SGs in the pathogenesis of PolyQ disorders. This review aims at discussing the evidence supporting the existence of a link between SGs functionality and PolyQ disorders, by focusing on the biology of SGs and on the way it can be altered in a PolyQ disease context.

## Facts

- Stress granules (SGs) are non-membranous foci that assemble as one of the first responses to cellular stress, being composed by 40S ribosomal subunits, translation initiation factors, poly(A)^+^ mRNAs and RNA-binding proteins (RBPs).
- SGs functions include global translational arrest, biomolecules storage, mRNA triage and expression regulation, cell signalling and apoptosis, and viral replication inhibition.
- RBPs are the main components of SGs and are crucial for neuronal gene expression regulation, which is highlighted by the fact that 50% of known RBPs are expressed in the brain.
- SGs co-localize with the pathological protein aggregates often associated with neurodegenerative diseases, including amyotrophic lateral sclerosis, frontotemporal lobar degeneration, and Alzheimer’s disease.
- Polyglutamine diseases are neurodegenerative disorders caused by proteins that bear an abnormally expanded glutamine stretch and that form insoluble aggregates.

## Open questions

- Can any parallels be drawn between SGs assembly and the multiprotein aggregation observed in polyglutamine diseases?
- Can SGs assembly dynamics be explored as a therapeutic target in the context of polyglutamine diseases?
- Does ataxin-2 or any other SG-composing RBP hold a particular role in the pathophysiological network of polyglutamine spinocerebellar ataxias that could help explain the similarities existing between these diseases?
- Is the involvement of SGs in polyglutamine diseases cytotoxicity mechanisms related with the regionally selective neurodegeneration profiles observed in these disorders?
- How do SGs deal with the CAG-expanded mRNAs that are generated in the context of polyglutamine diseases, and that have been described to be toxic even when untranslated?

## Introduction

Cells are repeatedly exposed to different stress stimuli during their lifetime. Stressing factors include conditions such as heat, nutrient shortage or hypoxia, as well as the dysfunction of particular cellular pathways or the accumulation of reactive oxygen species. Since cell stress affects homeostasis and normal cell functioning, stressing factors need to be overcome in order to ensure cell survival. Accordingly, cells utilize a wide range of mechanisms to cope with stress, involving, for example, transcriptional changes and alterations of protein degradation pathways, and eventually culminating in apoptosis in case they are unable to resist the aggressions.

One important set of mechanisms that cells use to overcome stress entails alterations in intracellular organization, through the formation of specialized membraneless compartments^1^. Stress granules (SGs) are one type of compartments whose assembly in the cytoplasm is essential for the cellular response to stress. SGs are known to contain a combination of ribosomal subunits, mRNA molecules and functionally diverse proteins, especially RNA-binding proteins (RBPs), but their exact functions have not been completely elucidated. Available evidence nonetheless indicates that SGs assembly constitutes an advantage in the context of cellular stress: (i) in these conditions, SG formation is more rapid than the transcriptional or translation changes brought about by the cellular stressors; (ii) important cellular molecules are protected from degradation upon SGs assembly during stress; and (iii) SGs disassembly after stress relief allows cells to have proteins and mRNAs ready to be used^2^.

While SGs are essential for normal cell functioning and to their adaptation to suboptimal environmental conditions, there is growing evidence suggesting that a persistent cellular stress state, in which SGs are also persistent, may underlie an enhanced susceptibility to aging or aging-related diseases, including neurodegenerative disorders (NDs) and cancer^3^. Additionally, in different NDs characterized by abnormal protein aggregation, not only are cells subjected to diverse sources of stress, but several stress–response pathways also seem to fail, possibly aggravating the pathology^4^. The current literature review aims to provide an updated outline of SGs biology and to explore and discuss the putative existence of a functional link between SG formation, a regulated process of biomolecule coalescence, and polyglutamine (PolyQ) diseases pathogenesis, often attributed to a contrastingly aberrant phenomenon of multiprotein aggregation and sequestration.

## What are stress granules?

When cells are subjected to a stress stimulus, one of their first responses is the formation of non-membranous organelles called stress granules (SGs). These cellular foci essentially consist of messenger ribonucleoprotein (mRNPs) complexes, in which mRNAs are stalled and translation inhibited, in order to save energy for the remainder components of the cellular response to stress^5^.

SGs were first described in tomato cell lines exposed to cellular stress induced by heat shock, and were thus initially designated as heat-stress granules (HSG)^6^. Through electron microscopy, the authors observed that the granular aggregates that were formed were highly enriched in heat shock protein 17 (Hsp17). Later, SG formation was also described in chicken embryo fibroblasts, wherein concentrated heat shock protein 24 (Hsp24) was detected in distinct insoluble aggregates found in the cytoplasm upon heat shock^7^. Since then, several studies demonstrated that SGs are formed in different in vitro models of diverse organisms, including plants, protozoans, fungi, *Caenorhabditis elegans* and mammalians, as well as in animal tissues and human patient samples.

**Fig. 1:**
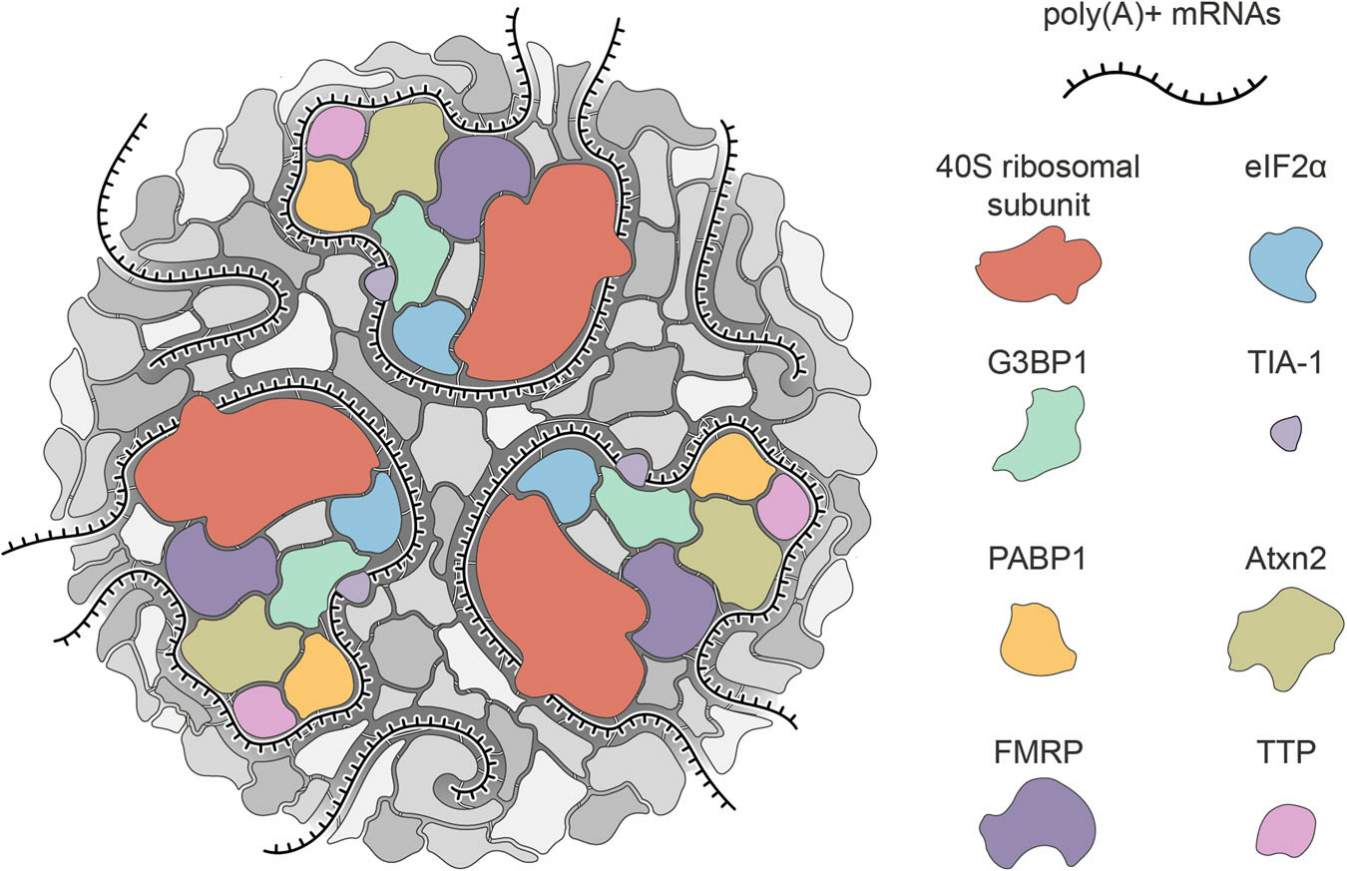
Stress granule components. Stress granules are multimolecular cytoplasmic foci that assemble as part of the cellular response to stress. They largely derive from stalled pre-initiation translation complexes and are mainly comprised of poly(A)^+^ mRNA molecules, 40S ribosomal subunits and a vast array of proteins (more than 450). The majority of these are RNA-binding proteins (RBPs) that bind to each other and to the other SG components. They include eukaryotic translation initiation factor 2 subunit alpha (eIF2α), ras GTPase-activating protein-binding protein 1 (G3BP1), T-cell intracellular antigen-1 (TIA-1), polyadenylate-binding protein 1 (PABP1), ataxin-2, fragile X mental retardation protein (FMRP) and tristetraprolin (TTP). The unspecified shapes coloured in grayscale represent the remaining proteins counted among the numerous SG protein components described so far.

SGs are mainly composed of stalled pre-initiation translation complexes: 40S ribosomal subunits, translation initiation factors, poly(A)^+^ mRNAs and RBPs^8^. Additionally, SG components also include protein kinases, RNA helicases, structural constituents of ribosomes, calcium-binding proteins, hydrolases and cytoskeletal proteins (Fig. 1). This complex and diverse composition of SGs suggests a notable variety of pathways and mechanisms in which these foci can be involved, and the crucial roles SGs assembly/disassembly has in the context of normal cellular function.

Importantly, SGs composition is different depending on the cellular context, as well as on the type of stressing factor causing their assembly, and its duration^9^. Taking this into consideration, our group has analysed and curated the available SG literature describing SG components detected in mammalian cells. To the best of our knowledge, we annotated all the protein components recruited to mammalian SGs that have been described so far, gathering them in an online open-access database (https://msgp.pt/)^10^. Currently, our database comprises 464 proteins identified as components of SGs, categorizing them according to their molecular functions or usual subcellular localization, and providing information about the type of cell or stressing condition in which they were detected.

### Stress granule formation and dynamics

A defining feature of SGs is their dynamism, as these foci quickly assemble upon stress induction and rapidly disperse when stressing conditions abate. The assembly and composition of SGs depend on the type of cells in question, the particular stressing factors involved and the signalling pathways that are activated. SG formation can be triggered by different conditions, such as endoplasmic reticulum stress, oxidative stress, heat shock, hypoxia, starvation, presence of translation-blocking drugs, viral infection, knockdown of specific translation initiation factors and overexpression of specific RBPs^11^.

**Fig. 2:**
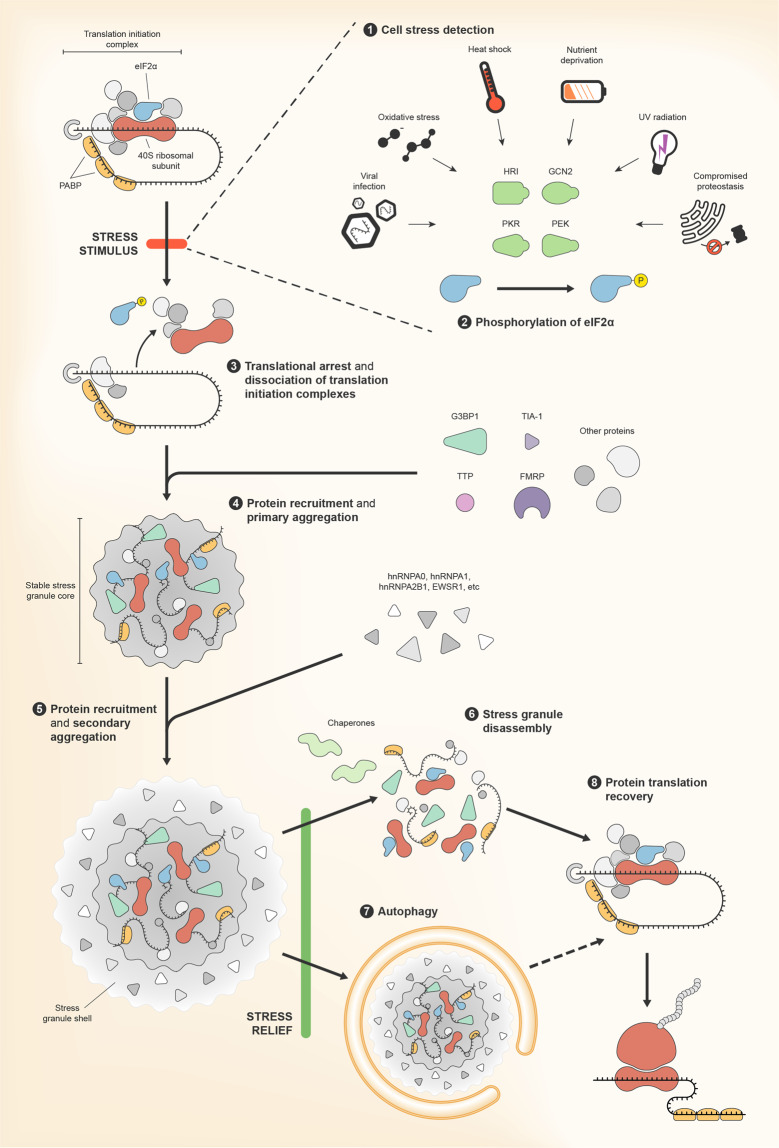
The canonical stress granule assembly pathway. (1) Formation of stress granules (SGs) can be triggered by diverse cell damaging conditions, including viral infection, oxidative stress, heat shock, nutrient deprivation, ultraviolet radiation or proteotoxic stress. Particular stress conditions are detected by specific kinases—protein kinase R (PKR), haem-regulated inhibitor (HRI), general control non-derepressible-2 (GCN2) and pancreatic eIF2α kinase (PEF)—that then become activated and (2) phosphorylate eukaryotic translation initiation factor 2 subunit alpha (eIF2α). (3) eIF2α is involved in the formation of translation initiation complexes and, when phosphorylated, leads to dissociation of these complexes and to translational arrest. (4) mRNAs, 40S ribosomal subunits, and proteins involved in translation start to accumulate and to assemble together, along with other proteins that are recruited to the forming SGs. This primary aggregation process produces a stable SG core. RNA-binding proteins (RBPs) that constitute SG cores include ras GTPase-activating protein-binding protein 1 (G3BP1), T-cell intracellular antigen-1 (TIA-1), tristetraprolin (TTP) and fragile X mental retardation protein (FMRP). (5) A secondary aggregation step resulting from additional, albeit weaker, intermolecular interactions originate the shell of the SGs. RBPs recruited in this step include heterogenous nuclear ribonucleoprotein A0 (hnRNPA0), hnRNPA1, hnRNPA2B1 and RNA-binding protein EWS (EWSR1). (6) When stress conditions abate, SGs are either disassembled by molecular chaperons or (7) are cleared by autophagy. (8) Disassembly allows for a rapid recovery of protein synthesis.

In the canonical SGs assembly pathway (Fig. 2), the first step is the phosphorylation of eukaryotic translation initiation factor 2 subunit alpha (eIF2α)^12^ by one of the four kinases: general control non-derepressible-2 (GCN2, or eIF2α kinase 4 (EIF2AK4)); pancreatic eIF2α kinase (PEK; alternatively PKR-like ER kinase (PERK) or eIF2α kinase 3 (EIF2AK3)); protein kinase R (PKR) or haem-regulated inhibitor (HRI); or eIF2α kinase 1 (EIF2AK1)^13^. The structure of each of these kinases contains specific regulatory regions that detect and recognize different stress stimuli. Phosphorylation of eIF2α leads to translational arrest and dissociation of translation initiation complexes from polysomes^8^. This results in the accumulation of mRNAs, translation factors, RBPs and other proteins that have intrinsically disordered domains (IDDs) or prion-like domains (PLDs)^14^. These regions are low-complexity sequences enriched in glycine and uncharged polar amino acids (serine, asparagine and glutamine), and often punctuated by aromatic (like tyrosine and phenylalanine) or charged residues^15,16^. They promote numerous electrostatic interactions between different regions of a protein, and between different proteins, giving rise to two regionally distinct states within the assembling granules: a stable core, surrounded by a dynamic shell^16^.

Core components of SGs—including nucleating RBPs such as ras GTPase-activating protein-binding protein 1 (G3BP1), T-cell intracellular antigen-1 (TIA-1), TIA-1-related (TIAR) protein, tristetraprolin (TTP), and fragile X mental retardation protein (FMRP)—bind to each other, as well as to polyadenylated mRNAs and 40S ribosomal subunits^17^, and induce the recruitment of other proteins, initiating the process of aggregation in the cytoplasm (Fig. 2). This event is denominated as primary aggregation and results in the formation of the stable core. In the secondary aggregation event, SG nucleators induce homotypic and heterotypic interactions between different SG components, resulting in the maturation of SGs and forming the shell, where numerous dynamic, and weaker, protein interactions occur. Proteins recruited to the *foci* include secondary RBPs such as heterogenous nuclear ribonucleoprotein A0 (hnRNPA0), hnRNPA1, hnRNPA2B1, RNA-binding protein EWS (EWSR1)^18^ and ataxin-2^19^. This mature state produces a liquid–liquid phase separation, i.e. the weak, but numerous, intermolecular interactions established between the RBPs generate a fluid droplet in the core of the aqueous cytosol, constituting a non-membranous organelle^18,20^. This type of phase separation of biomolecules, commonly involving proteins containing IDDs, is currently regarded as a fundamental and ubiquitous aspect of subcellular compartmentalization, playing critical roles in many cellular processes. In the case of SGs, the dynamic nature of the phase separation allows the transition of several proteins and RNA remodelling complexes between the core and the shell of the SGs, and even between these foci and the surrounding cytosol^21^.

A non-canonical pathway of SG assembly, independent from eIF2α phosphorylation, has also been reported^22^. It has been shown that inhibition of the eukaryotic initiation factor 4F (eIF4F) complex prevents the formation of the 48S initiation complex, inhibiting ribosome activity and arresting translation initiation. These events lead to SG formation, independently from eIF2α phosphorylation^22^.

SGs rapid dissipation upon stress removal allows the recycling of their components for immediate cellular use, in a process that is regulated by molecular chaperons (Fig. 2)^1^. In neurons, autophagy also seems to be implicated in SGs disassembly^23^, although it may not be the preferred pathway for their clearance^24^. SGs disassembly correlates positively with a recovery in overall protein synthesis and with the translation of several mRNAs^25^.

### Stress granules functions

**Fig. 3:**
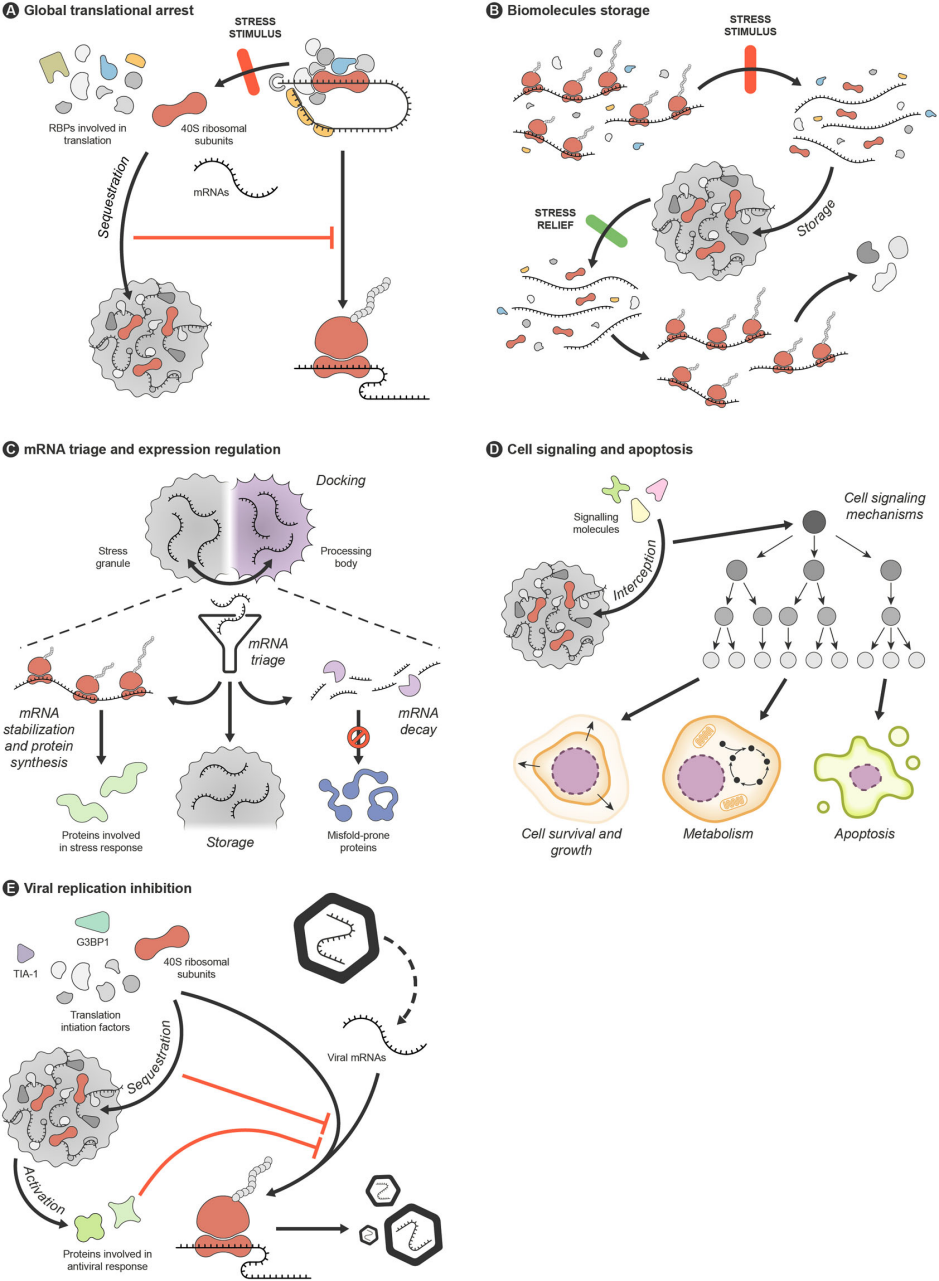
Stress granules functions. Stress granules (SGs) participate in the cellular response to stress through a set of different actions that are interconnected and derive from the individual activities of SG components and from the assembly/disassembly of these foci. **A** SG formation involves the disassembly of translation initiation complexes and the coalescence of mRNAs molecules, ribosomal subunits and many proteins involved in translation, resulting in translational arrest and protein synthesis suppression. Additionally, several SG components are known to act as translational repressors. **B** SGs function as stores of RNAs and proteins in cells under stress, but allow rapid mobilization of these molecules when the damaging conditions subside. **C** SGs may modulate the expression of specific proteins during stress, by directing particular mRNA species to different possible fates. This action appears to involve an exchange of mRNAs between SGs and processing bodies, upon docking of these two types of RNA granules. Translation of some proteins involved in stress responses may be prioritized, some mRNAs may be kept stored in SGs or elsewhere, while others, such as those codifying proteins prone to misfolding, may be targeted for degradation by the processing bodies. **D** By intercepting particular signalling molecules, SGs may trigger signalling cascades that regulate or modify cell growth, survival or metabolism, or which promote apoptosis. **E** SGs play a role in the cellular response against viral infection, by sequestering the endogenous translational machinery necessary for viral protein expression and by activating proteins involved in antiviral response.

Classically, SGs have been understood to serve as cellular storage foci responsible for translational arrest during a stress event^26,27^. Currently, they are also admitted to play a more active role in the stress response, participating in mRNA triage, stress signalling and apoptosis induction, among other processes. All of these functional roles are at least partially interconnected, and largely derive from the interplay between the individual functions of the diverse proteins and mRNA species that gather and coalesce into the SGs (Fig. 3).

#### Global translational arrest

The first role proposed for SGs was that of effectors of stress-induced translational arrest^28^. During stress, and upon phosphorylation of translation initiation factor eIF2α, SGs are assembled and translation is arrested, suggesting that these foci have functions connected with mRNA translation and localization^28^. For example, during cold shock-stress, global protein synthesis is suppressed and SGs are assembled, both ensuring cell survival during hypothermia^29^.

It has been hypothesized that transiently formed SGs function as foci where RNA translation is repressed and reprogramed under stressful conditions^27^. The fact that small ribosomal subunits, translation initiation factors, different signalling molecules and other players involved in translation besides RBPs are also assembled into SGs^2^ strengthens the idea that SGs may be crucially active effectors of translational repression. Moreover, it has also been suggested that SGs may be integrated with miRNA-induced translational silencing pathways^30^.

Additionally, SG formation is positively correlated with a decrease in global translation levels^2^ and several SG components are known to function as translational regulators. For example, a study has shown that the ablation of ataxin-2 leads to a reduction in the global translation rate^31^. The assembly of proteins (mainly RBPs) and mRNAs in SGs may prevent these components from integrating the translational machinery, thus contributing to the repression effect^2,27^.

It is not completely clear if SGs assembly per se is important for the translational repression of certain mRNAs and further studies are needed to clearly establish SGs definitive role in these mechanisms. In fact, some studies have suggested that SG formation is not essential for global translation repression during stress^32^ and that the impairment of SGs assembly by depletion of core factors does not affect global protein synthesis^33^.

#### Biomolecules storage

In line with the SGs proposed role in translational arrest is also their function of storage. Since de novo protein synthesis is an energetically expensive process for the cell, SGs constitute transient storage sites for mRNAs released from disassembled polysomes during stress. This confers an advantage to the cells that form SGs, from an energetic point of view. While SGs are forming, many mRNAs, such as those corresponding to housekeeping genes, are selected to be stored in these foci^2,34^. When the stressful period ceases, SGs are disassembled and the stored mRNAs can either move back to polysomes to restart translation, or be degraded in processing bodies^34,35^.

#### mRNA triage and expression regulation

SGs have consequently been implicated in the mRNA triage process^34,35^, through which mRNA molecules from disassembling polysomes are sorted and the fate of individual transcripts is determined. Supporting this hypothesis, evidence has shown that, during arsenite-induced stress^36^, mammalian SGs dock with processing bodies (PBs), which constitute another type of cytoplasmic foci that has been implicated in mRNA storage and, especially, decay. While docking, SGs and PBs share many protein components, including Fas-activated serine/threonine kinase (FASTK), 5ʹ−3ʹ exoribonuclease 1 (XRN1), eukaryotic translation initiation factor 4E (eIF4E), TTP and butyrate response factors 1 and 2 (BRF1and BRF2)^36^, as well as several mRNA species^37^. In the presence of environmental cellular stress, both PBs and SGs are simultaneously assembled and, through the exchange of components, SGs may prioritize the translation or degradation of some mRNA transcripts over others, thereby altering the proteome until stress conditions subside. Whereas specific transcripts are selected for decay by destabilizing proteins (e.g. TTP), other transcripts are bound by stabilizing proteins (e.g. Hu-antigen R (HuR), otherwise known as ELAV-like protein1, ELAVL1) for transport or storage, within the SGs or elsewhere^27^. SGs assembly may help cells prevent the accumulation of misfolded proteins by reducing the synthesis of certain transcripts, while optimizing the translation of mRNAs involved in stress response. For example, several mRNAs encoding proteins involved in stress response, like heat shock proteins, are excluded from SGs^36^.

It has also been proposed that SGs may promote destabilization of mRNAs by conditioning the activity of several stabilizing proteins, like HuR or zipcode-binding protein-1 (ZBP1), therein contained^25,32^. The recruitment of these proteins to SGs limits their cytoplasm availability, and thus their function. For example, it was shown that ZBP1 knockdown induced a selective destabilization of its target mRNAs^38^.

Some studies have challenged this view of SG’s role in mRNA triage, suggesting that SGs are not required for mRNAs destabilization during stress^39,40^. For example, one study showed that the stabilization of bulk mRNA including ZBP1 target transcripts is largely independent of SG formation during a stress stimulus^40^.

#### Cell signalling and apoptosis

In cells undergoing stress there is an interplay between diverse signalling pathways and SG formation. SG formation communicates a “state of emergency” through the interception of subsets of signalling molecules, which in turn triggers a cascade of signalling events that regulate SGs assembly. The localized enrichment of proteins in SGs may either serve to increase the rate of biochemical reactions within these foci or to reduce the cytosolic concentration of specific proteins by sequestration, both resulting in the alteration of several cell signalling pathways^41^.

By modulating several signalling pathways during stress, SGs interfere with cell survival, metabolism and growth. In particular, SGs assembly and dynamics may be important in the cellular decision to undergo apoptosis or not, depending on the response to stress. Several apoptosis regulatory factors are recruited to SGs, possibly inhibiting or delaying stress-induced cell death signalling^41^. For example, during severe, apoptosis-inducing, stress, the receptor of activated protein C kinase 1 (RACK1) protein binds to stress-responsive MAP 3 kinase (MTK1) and facilitates its activation; during modest stress RACK1 is recruited to SGs, limiting MTK1 kinase activation and avoiding apoptosis^42^. The recruitment of regulatory-associated protein of mTOR (Raptor) to SGs also prevents the overactivation of mTORC1 signalling, thus inhibiting apoptosis^43^. Additionally, in stressed cells, SG formation reduces the production of reactive oxygen species, thereby also preventing apoptosis^44^. Also in line with SGs participation in cell survival decisions, other studies reported that impairing SGs assembly leads to a decrease in cell viability after stress exposure^45^, and that inhibiting SG formation by oxidizing TIA-1 made cells more vulnerable to apoptosis^46^.

The role that SGs have in cellular growth appears to involve regulation of the mTOR pathway, which monitors nutrient levels and energy availability to promote either cell growth, when conditions are favourable, or catabolic processes, during stress^29,43,47^. For example, the dual specificity tyrosine-phosphorylation-regulated kinase 3 (DYRK3), which is recruited to SGs, phosphorylates the mTORC1 inhibitor proline-rich AKT1 substrate 1 (PRAS40), allowing mTORC1 to exit SGs in an activated state and to fulfil its signalling functions in cell growth and metabolism^47^.

#### Viral replication inhibition

SGs can impact virus replication and force viral adaptation by sequestering and binding cell components as part of their role in translational arrest and RNA decay. For example, TIA-1, TIAR (ref. ^48^), G3BP1 (ref. ^49^), translation initiation factors and the 40S ribosome subunit—all of them components of SGs—are required for the replication of any virus. Additionally, SGs that are induced during viral infection recruit many innate immune proteins, and several studies have revealed that SGs serve as a platform for activation of protein kinase R (PKR) and the retinoic acid-inducible gene I (RIG-I) dsRNA helicase, which are both essential to initiate antiviral response^50,51^. Thus, SGs can have inhibitory effects on viral replication, acting as players in the antiviral response^52^. Conversely, several reviews describe that different virus interfere with SG formation and regulation, consequently affecting the cellular functions of these granules^52^.

## RNA-binding proteins, stress granules and human disease

So far, more than 1000 mammalian genes have been identified as coding for RBPs, and 20% of all known proteins are RBPs^53^. In line with this, and as mentioned above, RBPs are one of the main components of SGs: from the 464 proteins identified as SG components^10^, 252 (54%) are classified as RBPs^54^. RBPs have low-complexity domains, that make them prone to aggregation and facilitate protein-protein interactions, possibly explaining their high prevalence in SGs^18^.

RBPs are involved in different steps of RNA metabolism and thus control many aspects of an RNA molecule life cycle in the cell. These functions of RBPs vary according to their subcellular localization. For example, in the nucleus, RBPs have been implicated in functions such as the regulation of mRNA maturation, RNA polymerase elongation and nuclear export^55^. In the cytoplasm, RBPs seem to be involved in the regulation of RNA transport, silencing, translation and degradation^56^. The wide range of functions performed by RBPs are mainly due to the multiplicity of their interaction domains, which allow them to interact with many different proteins^57^.

Being essential for a wide variety of cell functions, ranging from the regulation of gene expression to post-transcriptional processes, it is not surprising that dysregulation in the expression of different RBPs has been suggested to underlie several human disorders, including cancer and NDs^58,59^. Similarly, perturbations in the assembly or localization of SGs, which are supported by RPBs, may be involved in different human conditions including cancer^60^, aging^11^ and NDs^8^.

For example, it was found that at least 30 different RBPs were upregulated in different types of cancers. The authors of this study proposed the idea that fluctuating RBP levels could result in the increase of non-specific protein interactions with an important impact on the disease outcome^61^. In human breast cancer biopsies, cancer cells sometimes accumulate the metastatic lymph node gene 51 (MLN51) RBP, in discrete cytoplasmic foci resembling SGs^45^, which is in line with studies that have associated SG formation to the resistance to chemotherapeutic drugs^62^.

It has also been found that the expression of several RBPs is reduced in aged individuals, suggesting that they could play important roles in maintaining tissue homeostasis with advancing age^63^.

### RBPs, stress granules and neurodegeneration

More than 50% of known RBPs are expressed in the brain, where they are involved in different processes such as alternative splicing, transport, localization, stabilization and translation of RNAs^64^. Importantly, RBPs are components of neuronal RNA granules, also called transport ribonucleoprotein particles (RNPs), which are motile structures that transport mRNA and contain several translational components^65^.

Given the important role of RBPs for cellular function, it is expected that their dysregulation may have a profound effect in neuronal health, contributing to the disruption of different pathways underlying the pathogenesis of NDs. In fact, several studies have shown that different SG-nucleating RBPs are associated with neuronal defects. For example, it has been shown that depletion of SG core component G3BP1 in neurons leads to an increase in intracellular calcium and calcium release. This implicates G3BP1 in the control of neuronal plasticity and calcium homeostasis, establishing a link between SG formation and neuronal dysfunction^66^.

The possible link between NDs and SGs is strongly supported by evidence of co-localization between these foci and the pathological protein aggregates often associated with degenerative diseases of the nervous system. Different SG components are present in the pathological protein aggregates of conditions that include amyotrophic lateral sclerosis (ALS), frontotemporal lobar degeneration (FTLD) or Alzheimer’s disease (AD)^67^. The possible implication of SGs in the context of NDs has been explored in recent years, especially in the case of AD and ALS (refs. ^18,68^). It was shown that SGs assembly in neuronal cells promotes the formation of phosphorylated tau inclusions and, likewise, tau seems to stimulate SG formation^69^. Another study found that the chronic exposure to the amyloid-beta peptide stimulates the formation of persistent SGs, which are also found in patients with severe AD (ref. ^70^). Moreover, in AD, several SG components accumulate in affected cells and co-localize with pathogenic tau^69,71^. In ALS, SG components such as TIA-1, eukaryotic translation initiation factor (eIF3) or polyadenylate-binding protein (PABP) co-localize with neuropathology markers in patients’ brain^72^. The accumulation of TAR DNA-binding protein 43 (TDP-43) inclusions was shown to be associated with SGs, the two co-localizing in degenerating neurons^72^. In fact, different studies showed that prolonged SG formation may contribute directly to ALS (ref. ^73^).

The possible role of SGs in the pathogenesis of NDs is also highlighted by the fact that mutations or malfunctions in the genes encoding for different SG components are the direct cause of some of these disorders or are closely implicated in pathogenesis, in other cases. For example, abnormal expansions in FMRP cause fragile X mental retardation syndrome (FXS), mutations in Survival motor neuron 1 (*SMN1*) are linked to spinal muscular atrophy (SMA), while mutations in TDP-43, FUS, optineurin (OPTN), and angiogenin (ANG) cause motor neuron diseases, including ALS (ref. ^18^). Mutated TDP-43, the major pathological protein in sporadic ALS, is associated with the inactivation of 5ʹAMP-activated protein kinase (AMPK), which is induced by energy depletion and metabolic stress, thus impacting the outcome of ALS (ref. ^74^). Recently, Sleigh and colleagues also reported that mice expressing mutated TDP-43 exhibited a deficit in axonal transport of signalling endosomes, contributing decisively to the neuropathology observed in the disease^75^. Mutant expansion in the CAG tract of ataxin-2 causes spinocerebellar ataxia type 2 (SCA2), and intermediate size expansions in the same protein are a risk factor for ALS (ref. ^76^). A loss-of-function mutation of LIN28A in patient-derived human embryonic stem cells / induced pluripotent stem cells and in a mouse model contributes to Parkinson’s disease (PD) pathology^77^.

Altogether, there is important evidence suggesting a major role for SGs in the pathogenesis of NDs.

## RBPs, stress granules and polyglutamine disorders

Polyglutamine (PolyQ) disorders constitute a group of hereditary NDs, which includes Huntington’s disease (HD), spinal and bulbar muscular atrophy (SBMA), dentatorubral-pallidoluysian atrophy (DRPLA) and six types of spinocerebellar ataxia (SCAs). These disorders are caused by an abnormal expansion of CAG triplets in the open reading frame of the causative genes, which encode for an expanded PolyQ tract in the respective proteins. The different PolyQ-causing proteins are otherwise unrelated and display no significant similarity besides the PolyQ tract; it is this common feature—the PolyQ tract—that, when expanded, affects their local and global protein structure and promotes abnormal self-assembly^78^. PolyQ protein aggregation culminates in the formation of multiprotein, macromolecular, intracellular inclusions, which are a key histological feature of PolyQ disorders.

The toxic nature of the molecular species that are formed during the aggregation process is still a matter of debate, and it remains unclear whether aggregation is the cause, or the consequence of the progressive neurodegeneration observed in PolyQ disorders. It has been suggested that the formation of expanded PolyQ-containing aggregates in the nucleus and/or the cytoplasm occurs before other cell defects are detected^79^. In fact, expanded PolyQ-containing proteins are known to have a tendency to spontaneously self-assemble, even in vitro, forming insoluble aggregates^80^. On the other hand, some authors have suggested that aggregates, and especially the macromolecular inclusions observed in post-mortem patients’ brains, may represent an end-stage manifestation, posterior to the toxicity events leading to neurodegeneration^81^.

Current evidence it still insufficient to draw a clear picture of the events linking expanded PolyQ protein expression and disease, but, despite all the uncertainties, the tendency of expanded PolyQ-containing proteins to engage in aberrant interactions and aggregate have been repeatedly pointed as factors responsible for cell toxicity in the context of PolyQ disease pathogenesis. Aggregation disturbs protein-folding homeostasis and alters the solubility and localization of other proteins^82,83^. PolyQ protein aggregates display a tendency to sequester functionally diverse proteins and thereby disrupt the cell systems with which they are engaged^84^. In particular, this phenomenon may compromise transcription, protein quality control and intracellular transport, as a result of the documented sequestration of transcription factors, chaperons, proteasome components and motor proteins^85–87^. As an example, it was shown that expansion of the PolyQ segment of huntingtin (Htt) beyond the pathological threshold for HD resulted in the structural perturbation of an adjacent (fused) β-barrel protein, increasing the propensity of both to aggregate^88^.

The pathological protein aggregation process occurring in PolyQ disorders starkly contrasts with the regulated and functional protein coalescence observed in SGs assembly. However, RBPs may be especially susceptible to the multiprotein aggregation cascades triggered by expanded PolyQ, given the fact that they contain low-complexity domains and have some propensity to aggregate per se^80,89,90^. Moreover, mutations in several SG components are known to increase their propensity to aggregate and to induce SG formation^8^.

It is thus logical to envision that the increased tendency of PolyQ proteins to aggregate and abnormally interact with other proteins may promote the recruitment of SGs, or at least some of their components, into pathological protein agglomerates. This may affect the normal functions of those components, and compromise SGs action in countering cell stress. Additionally, protein sequestration, along with a multifactorial and permanent state of cell stress caused by PolyQ-expanded protein expression, may alter SGs dynamic assembly and disassembly process, again compromising their biological roles. The abnormal SGs that are formed as part of a chronic stress response may themselves constitute a source of further cell toxicity. The following sections discuss the hypothesis whereby SGs dysfunctionality, caused by these diverse, albeit related, events, plays a pivotal role in the pathogenesis of PolyQ disorders.

### Sequestration of stress granules components into PolyQ aggregates

It has been demonstrated that proteins with very long intrinsically disordered domains (IDDs), which are frequent in RBPs, are particularly vulnerable to be recruited into PolyQ aggregates^91^. In particular, Ratovitski and colleagues^92^ have shown that expanded huntingtin preferentially interacts with proteins containing IDDs. Aggregates formed by PolyQ-containing proteins have been described to be β-rich fibrillar structures of amyloid nature, which induce cytotoxicity by sequestering components of quality control systems and transcriptional machinery; this type of aggregates has been shown to interact with functionally diverse RBPs^87,90,93,94^.

In fact, studies show that SG components are usually found in the neuropathological protein aggregates that are characteristic of PolyQ diseases^76,95,96^. For example, it was demonstrated that TIA-1 co-localizes with perinuclear mutant Htt aggregates, in cell cultures^97^, and with the Htt mutant aggregates of a HD mouse model^95^. In SCA2 patients, TDP-43 co-localizes with pathological inclusions of mutant ataxin-2, itself a SG component^76^.

In transfected cells, aggregates of an Htt fragment in the cytoplasm caused the sequestration and mis-localization of proteins containing disordered and low-complexity sequences, significantly impacting their nucleo-cytoplasmic transport^94^. Concordantly, mRNA abnormalities were detected in HD transgenic mice: a fraction of analysed neurons presented either mRNA nuclear accumulation or an overall reduction of mRNA levels.

### Cell stress in PolyQ diseases

While the molecular and cellular physiopathology of PolyQ diseases still poses many unanswered questions, it is increasingly clear that it involves the dysfunctionality of several cell systems. Accordingly, current evidence suggests that cells affected by PolyQ toxicity are subjected to cell stress from more than one source. In particular, it has been reported that PolyQ-expanded protein expression triggers proteotoxic and oxidative stress^98,99^.

Correct folding of proteins and maintenance of their structural integrity is essential for cell homeostasis. PolyQ-expanded proteins pose challenges to the mechanisms responsible for proteostasis, as they are prone to aggregate and to engage in abnormal intermolecular interactions. Proteotoxic stress is associated with a collapse of the protein quality control mechanisms, namely those involving the activity of molecular chaperones and protein-degradation systems^98,100^. These mechanisms have been described to be impacted by PolyQ protein expression: for example, chaperones and/or proteasome components have been detected in protein aggregates associated with HD, SCA1, SCA3 and SBMA, in cell models^101–103^, animal models^104,105^ and patient brain samples^106,107^; autophagy has been described to be impaired in animal models of SCA1 and SBMA (refs.^108,109^), and in patients of HD, SCA3, SCA7 (refs. ^110,111,112^). Endoplasmic reticulum stress, translated as an accumulation of proteins of the secretory pathway in this cell compartment, may also constitute a facet of proteotoxic stress in PolyQ diseases^113^. It has been suggested to be a consequence of the endoplasmic reticulum-associated degradation impairment that has been observed to arise upon expression of expanded PolyQ proteins^114^. For example, in SCA3, it was shown that changes in the interaction between ataxin-3 and valosin-containing protein (VCP/p97) leads to dysfunctions in ataxin-3 function as a regulator of endoplasmic reticulum-associated degradation^115^. Imbalances in the misfolded protein load in the endoplasmic reticulum may constitute a source of proteotoxic stress.

One of the targets of PolyQ protein toxicity is mitochondrial function^99,116,117^. In experimental models of several PolyQ diseases, including HD, SCA3 and SCA2, changes in these mitochondria have been associated with the disruption of the redox equilibrium and the production of reactive oxygen species, the effectors of oxidative damage to biomolecules^118,119^. Importantly, levels of oxidative stress markers have been described to be increased in HD patients’ brain^120^ and in blood samples from HD (ref. ^121^) and SCA3 patients^122^. For example, in fibroblasts of SCA2 patients, it was found that expanded ataxin-2 interacts with NADPH oxidase membrane subunit gp91, activating its enzymatic activity^123^. This triggers a signalling cascade that appears to be responsible for the generation of an oxidative wave that induces mitochondrial stress, DNA damage, and inhibition of neural-specific transcription.

These and other sources of cell stress may contribute to a state of chronic stress in cells affected by PolyQ protein toxicity. Adding to the direct injuring impact that the stressors have on affected cells, PolyQ protein toxicity may render some stress response systems dysfunctional and lead to a state of increased vulnerability to stress^124^. Surmounting evidence does suggest that stress response is aberrantly altered in the context of PolyQ diseases. Molecular chaperone heat shock protein 70 (Hsp70) was shown to be downregulated in a HD mouse model, and this effect was suggested to result from transcription factor NF-Y sequestration by mutant Htt^125^. Ataxin-3 has been shown to activate transcription factor forkhead box protein O4 (FOXO4) and induce the expression of manganese superoxide dismutase (SOD2), an antioxidant enzyme, under oxidative stress conditions; however, when ataxin-3 is expanded, this transcriptional activation effect is reduced and, in fact, SOD2 levels are decreased in SCA3 patients brain samples^126^. Levels of several antioxidant molecules, including cysteine, glutathione and ascorbic acid have been described to be lowered in HD (ref. ^116^). Expression of cystathionine γ-lyase, an enzyme involved in cysteine biosynthesis, was shown to be decreased in HD patient brain samples^127^. The team that produced these observations further suggested that this effect resulted from mutant Htt inhibitory effect on specificity protein 1, a transcriptional activator of cystathionine γ-lyase.

### Altered stress granule assembly and disassembly

A chronic state of cell stress may interfere with the normal functionality of the stress response mechanisms, including SGs. This abnormal activation of stress responses may, in turn, have a deleterious effect on cell survival. As mentioned, pathological aggregation of PolyQ proteins and the abnormal interactions in which they engage can result in significant changes in the protein clearance mechanisms and in the cellular stress response pathways^124,128^, possibly preventing formed SGs from disassembling and/or hindering SG assembly in response to stress.

The biological features of both the core and the shell of SGs can be altered during a stress response, and thus vary according to the duration of the stress stimulus. In an acute, transient, stress state, induced by conditions such as oxidative, metabolic, hypoxic, or thermal stress, SGs appear in the cytoplasm of the cells. Chronic SGs are fundamentally different from the ones assembled during acute stress conditions. Although only a limited number of reports regarding the composition of chronic SGs is available, this type of granules appears to act as a nidus for the aggregation of some disease-linked proteins. During persistent periods of stress, the phase-separated proteins of the shell can mature to become a gel-like layer, promoting aggregation, and turning into more stable complexes^18^. For example, it was reported that chronic SGs can recruit the ubiquitin-binding protein p62 and induce post-translational modification of RBPs by phosphate groups or ubiquitin^129^.

Interestingly, the prolonged coalescence of RBPs promotes the accumulation of β-sheet structured proteins, which can stack to form large macromolecular complexes^16,130^. Aggregated, β-rich fibrillar structures of amyloid nature have been repeatedly associated with diverse human conditions; not only PolyQ diseases, as mentioned above, but also with other NDs as well, including AD and PD. Stable amyloid aggregates containing proteins and RNA are suggested to play a central role in NDs, provoking the disruption of post-transcriptional changes^131^. Like Htt, a fragment of TDP-43, a SG component that forms detectable aggregates in ALS and frontotemporal dementia patients, was also described to cause protein mis-localization and RNA accumulation in the nucleus^94^. Cytotoxic amyloid aggregation in PolyQ diseases may thus result not only from PolyQ protein self-assembly, but also by the persistence of SGs induced by a chronic stress state.

One possible cause for imbalance of SG formation in PolyQ diseases concerns the autophagy pathway. It has been shown that enhanced autophagy activity reduces the number of SGs^132^. It was also shown that SGs are cleared by autophagy in mammalian cells, as their clearance is reduced by the inhibition of autophagy or by the depletion of VCP/p97, which is implicated in the pathway^5^. Later, it was shown that SGs are cleared by autophagy through the promotion of the formation of autophagosomes due to the recruitment of Syk kinase, another protein involved in autophagy, to SGs^133^. Autophagy has been demonstrated to be impaired in PolyQ diseases and in other NDs^112,134,135^. Thus, autophagy dysregulation may lead to the persistence of SGs, and the consequent sequestering of components thatate important in the cellular response to stress. In turn, this process may contribute to the consolidation of the pathological protein aggregates, since it was shown that normal SGs are free of misfolded proteins and autophagy players, whereas aberrant SGs contain misfolded proteins, which attract autophagy machinery components to SGs^24,136^.

### Ataxin-2 as a hub for PolyQ toxicity

Taken together, the evidence presented suggests the existence of a link between SGs dysfunctionality and the pathogenesis of PolyQ disorders, resulting from protein aggregation and sequestration. One protein in particular seems primed to play a central role in this toxic interplay of aggregation-prone proteins. Ataxin-2, the protein that causes SCA2 when its PolyQ tract is expanded beyond the critical threshold of 32 glutamine residues, is itself a component of SGs. Although its precise biological function is still unclear^19^, ataxin-2 is an RBP involved in SGs assembly, and its depletion strongly reduces the number of SG-positive cells upon stress induction^137^.

Ataxin-2 is present in the pathological protein aggregates of at least one other PolyQ disorder, SCA3 (ref. ^96^). What is more, we have shown that mutant protein aggregation in SCA3 is associated with a significant decrease in ataxin-2 mRNA and protein levels, as the reestablishment of ataxin-2 levels mitigates the neuropathological and behaviour deficits in different MJD/SCA3 mouse models^83^. Impairment of ataxin-2 functions in SCA2 and the other PolyQ diseases may produce an imbalance in SG dynamics and interfere with other cell systems with which ataxin-2 is involved; ataxin-2 interacts directly with mRNAs^138^ and with several proteins, either directly or indirectly^139,140^. In the case of SCA2, mutant ataxin-2 is able to alter mRNA stability, namely by an increase in transcripts levels, observed for STAU1, contributing to aberrant protein aggregation^141^. Other study reported that decreasing the expression of ataxin-2 reduced the aggregation of other RBPs, such as TDP-43, increasing cellular survival and attenuating the pathological process in TDP-43 transgenic mice^142^.

One of the puzzling features of PolyQ diseases is the fact that, although the genes and proteins that cause them are unrelated, when abnormally expanded all of them give rise to disorders affecting neuronal function and survival. This is particularly striking in the case of PolyQ SCAs, given the fact that they all involve a compromise of cerebellar structure and function and lead to partially overlapping clinical signs^143^. It has been suggested that genes and proteins involved in PolyQ SCAs and other hereditary ataxias may be functionally related, constituting the nodes of an intricate interaction network^144^. This network may be especially crucial for cerebellar cell function and survival and that would be why, when one of its members is disrupted, the whole network malfunctions, triggering common pathogenic mechanisms that lead to cerebellar ataxia. In line with this idea, for many years it has been known that many genes that are responsible for PolyQ diseases are themselves modifiers of other PolyQ proteins toxicity^145^. For example, in human SCA2 patients, alleles of SCA3 gene ATXN3 with longer CAG tracts predict an earlier age of onset^146^. If SG dysfunction is indeed a critical factor in PolyQ disease pathogenesis, it is tempting to propose that ataxin-2 may constitute an important nexus where the network of ataxia-related genes and proteins articulates with SGs machinery.

## Final considerations

Ever since SGs were first described, several studies have focused on investigating their assembly/disassembly dynamics, their cellular functions, the components recruited to these foci upon stress, and, more recently, their possible involvement in human disease. Considering that SGs are structures that result from a multiprotein assembly process, it seems relevant to look for the relation that SG formation may have with the aberrant protein aggregation observed in several human disorders such as PolyQ diseases, and the putative involvement of SGs in this process. SGs rapidly assemble upon stress, and when the stress stimulus is removed, they rapidly disappear. In contrast, in PolyQ disorders protein aggregation is a pathological and irreversible phenomenon.

**Fig. 4:**
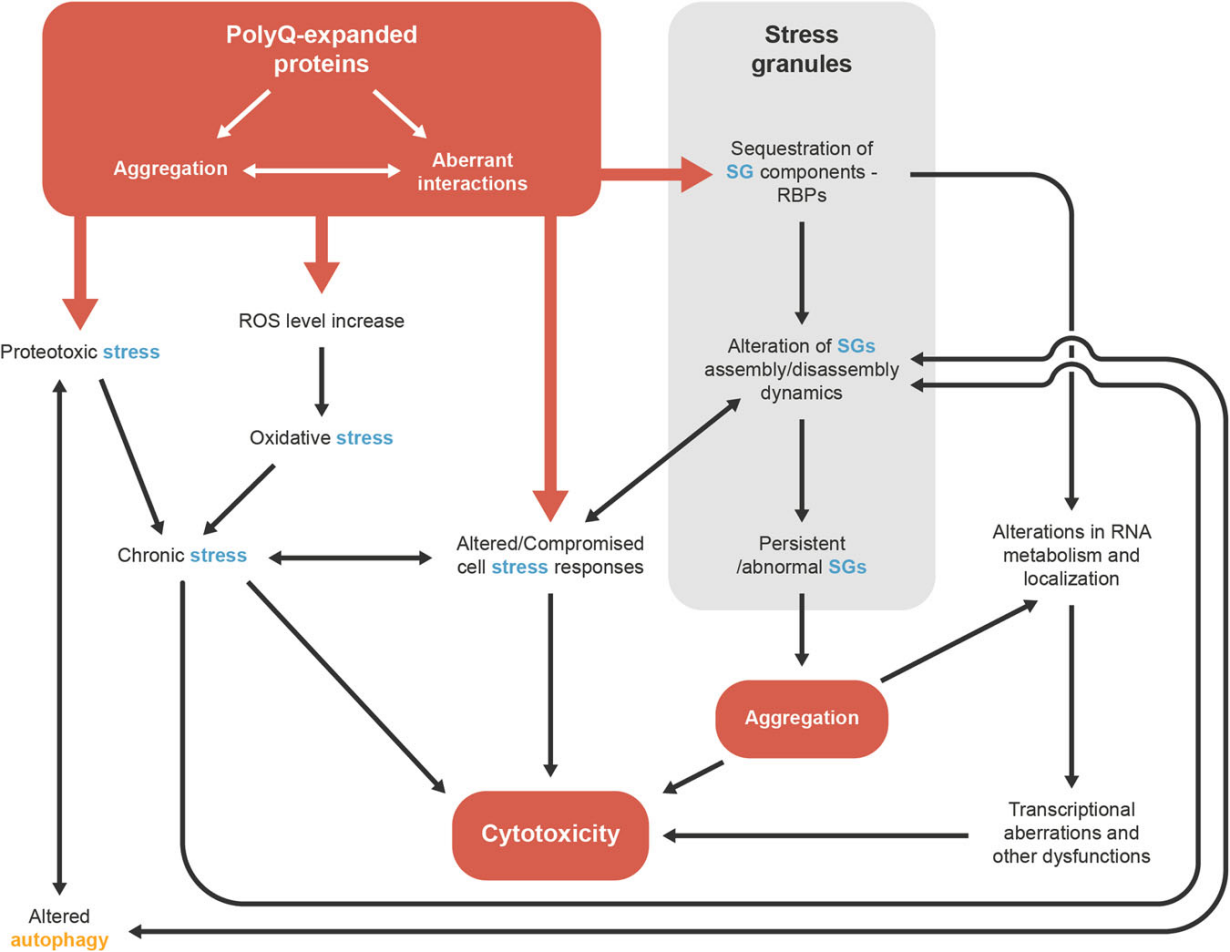
The putative involvement of stress granules in the molecular pathophysiology of polyglutamine diseases. Proteins bearing an expanded polyglutamine (PolyQ) tract display a tendency to aggregate and to engage in aberrant intermolecular interactions. Association of PolyQ-expanded proteins with stress granules (SGs) components, in particular RNA-binding proteins (RBPs) that are often prone to aggregate, may alter the dynamics of SGs assembly and disassembly. This can compromise SGs functionality, contributing to the globally deficient cell stress response that has been described to be a component of the molecular pathophysiology of PolyQ diseases. Oxidative stress (associated with an increase in reactive oxidative species - ROS - levels) and proteotoxic stress, which are known to result from expanded PolyQ protein expression, may culminate in a state of chronic cell stress, which may add to the abnormal SG assembly/disassembly dynamics and lead to the persistence of SGs. SGs formed under chronic stress are known to acquire abnormal properties and to seed toxic aggregation. Defects in autophagy caused by PolyQ protein expression may also contribute to the persistence of SGs and to their altered dynamics. Additionally, both the sequestration of RBPs to PolyQ aggregates and the toxic aggregation triggered by SGs may alter RNA metabolism and its subcellular localization, which in turn may lead to transcriptional aberrations. Chronic stress, a reduced ability to cope with cell stress, transcriptional alterations and a pernicious cascade of protein aggregation involving both the PolyQ-expanded proteins and the SGs may combine to produce the cytotoxic profile with is at the basis of cell dysfunction and loss, in PolyQ diseases.

SGs are mainly composed by RBPs, which are proteins that frequently contain low-complexity domains. These domains make RBPs more prone to aggregate, to interact with other proteins and to be recruited to aggregates. Inside cells, aggregate-prone RBPs normally participate in the repeating and highly dynamic cycles of functional assembly and disassembly of protein-RNA granules such as SGs^131^. On the other hand, the pathological expansion in PolyQ disorder-causing proteins causes them to aggregate abnormally and stably. Their presence may disrupt the normal RBP aggregation equilibrium; RBPs from SGs and abnormal PolyQ-containing proteins may produce an overactive aggregation phenomenon within neurons, which could underlie and decisively contribute to PolyQ disease pathogenesis. Sequestration of RBPs and other components of stress response systems may contribute to the transcriptional aberrations and the dysfunctions of other cell systems which are commonly envisioned as effectors of cytotoxicity in PolyQ diseases^99^. Cell stress, and stress response dysfunction, may further exacerbate abnormal protein coalescence, further feeding the aggregation cascade (Fig. 4).

PolyQ expansion and aggregation, possibly combined with cell stress, may not only decrease the ability of cells to eliminate the pathogenic protein but may also interfere with SG dynamics. SG’s disassembly in neurons seems to be linked to the autophagy pathway, which is dysregulated in several NDs, including PolyQ disorders. Autophagy impairment may contribute to the persistence of SGs, conditioning the availability of translational factors, mRNAs and other agents that are important for cellular function and that constitute SGs.

Current perspectives on the biology of proteins bearing low-complexity sequences suggest a putative mechanistic link between SGs biophysical properties and the pathologic aggregation of PolyQ-expanded proteins. Proteins enriched in low-complexity sequences are prone to form intracellular liquid-like condensates, of which SGs are an example^20^. Phase-separated membraneless organelles appear to be metastable, and while they can disassemble, they can also evolve into a solid-like state or they can directly drive formation of amyloid-like fibrils, as has been demonstrated for condensates of RBPs with low-complexity sequences that act as SG components^16^. PolyQ-containing protein aggregation has been long envisioned to be a nucleation-dependent process, whereby a critical monomeric expanded protein nucleus is initially formed, and other monomers are subsequently added to it down an energy gradient^89,147^. It has been proposed that the phase-separated, protein-rich, environment of the SGs may provide favourable conditions for amyloid-like fibrillization by increasing the probability of nucleation and the rate of monomer addition^16^. Physiological protein interactions between SG components and PolyQ-containing proteins may draw the latter to that environment, and in case they bear an aggregation-inducing PolyQ expansion, this may increase their tendency for pathogenic aggregation^16^. Consistent with this, liquid-like condensates of expanded Htt exon 1 have been shown to convert into fibrillar structures, in vitro and in cells^148^. While the process whereby liquid-like condensates may drive aggregation nucleation is not known, this environment seems to be also favourable for aggregate cross-seeding, i.e. for a particular protein to drive the aggregation of other proteins^149^. Functional coalescence of SG components and a possibly physiological fibrillization process^20^ may thus trigger pathogenic PolyQ protein cross- and self-assembly. Importantly, cross-seeding between different PolyQ-containing proteins or between a PolyQ protein and other amyloid-forming proteins (including prion amyloids) has been described^150^.

The idea that SGs play a relevant role in PolyQ diseases pathogenesis opens up a new avenue of research and interest in the field of PolyQ disorders, further linking it with the cellular mechanisms implicated in stress response. Future studies will contribute to a better understanding of the role of SGs in PolyQ disease pathogenesis, eventually leading to the identification of new molecular targets and to the development of new therapeutic strategies. The study of SGs functional aggregation may also constitute an interesting framework for the study of pathogenic protein aggregation, which plays a pivotal part not only in PolyQ diseases but also in many other human degenerative conditions.
